# Modulation of Temoporfin Distribution in Blood by β-Cyclodextrin Nanoshuttles

**DOI:** 10.3390/pharmaceutics13071054

**Published:** 2021-07-09

**Authors:** Ilya Yakavets, Igor Yankovsky, Tatyana Zorina, Mikhail Belevtsev, Lina Bezdetnaya, Vladimir Zorin

**Affiliations:** 1 Centre de Recherche en Automatique de Nancy, Centre National de la Recherche Scientifique, UMR 7039, Université de Lorraine, Campus Sciences, Boulevard des Aiguillette, 54506 Vandoeuvre-lès-Nancy, France; l.bolotine@nancy.unicancer.fr; 2 Research Department, Institut de Cancérologie de Lorraine, 6 Avenue de Bourgogne, 54519 Vandoeuvre-lès-Nancy, France; 3 Laboratory of Biophysics and Biotechnology, Faculty of Physics, Belarusian State University, 4 Nezavisimosti Avenue, 220030 Minsk, Belarus; iv.yankovsky@gmail.com (I.Y.); zorinate@mail.ru (T.Z.); vpzorin@mail.ru (V.Z.); 4 Belarusian Research Center of Pediatric Oncology, Hematology, and Immunology, 223053 Minsk Region, Belarus; belevtsev_m@mail.ru

**Keywords:** temoporfin, cyclodextrins, red blood cells, white blood cells, Raji human Burkitt’s lymphoma, flow cytometry

## Abstract

Photodynamic therapy represents a more targeted and less invasive alternative cancer treatment to traditional modalities. Temoporfin, as with many photosensitizers, is given by injection into a vein, and its subsequent fate is largely determined by the binding to plasma proteins and interaction with endothelial and blood cells. Thus, it is essential to be able to control and to alter the biodistribution of temoporfin in blood. In the present study, we evaluated the effect of co-administration of temoporfin with randomly methylated β-CD (Me-β-CD) on the distribution of temoporfin in the main subpopulations of blood cells of healthy donors using absorbance spectrophotometry and flow cytometry. We showed that cell-bound temoporfin fraction in blood strongly depends on the concentration of Me-β-CD. In fact, the accumulation of temoporfin in white blood cells was more sensitive than that in red blood cells, due to the higher volume of membranous organelles in white blood cells. Finally, we demonstrated that Me-β-CD significantly increases cellular uptake of temoporfin cancer human Burkitt′s lymphoma Raji cells. The presence of Me-β-CD resulted in a spotted pattern of temoporfin distribution in the plasma membrane compartment. Our results clearly demonstrated that β-CDs derivatives provide new options to modulate temoporfin biodistribution in blood.

## 1. Introduction

Photodynamic therapy (PDT) is an alternative cancer treatment offering a more targeted and less invasive treatment regimen compared to traditional modalities. Photodynamic therapy (PDT) employs a combination of the photosensitizer (PS), light, and molecular oxygen to selectively target tumor cells via cytotoxic activity [1]. One of the chief benefits of PDT compared to other cancer treatment modalities is the dual selectivity due to the selective PS accumulation in the target tissue and light delivery in a spatially confined and focused manner [2,3]. PDT-based cancer therapy is especially beneficial to patients in whom size and/or tumor location limits the application of conventional therapy.

**Figure 1:**
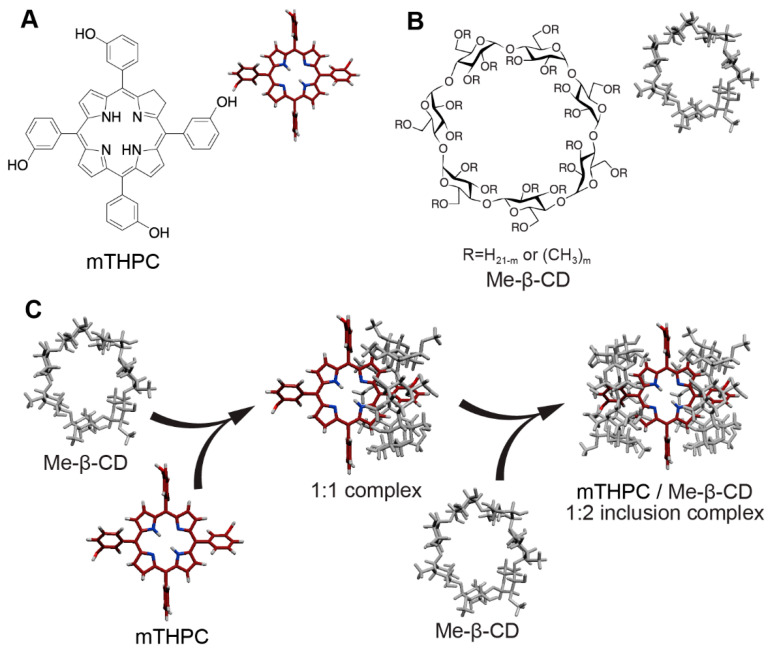
Molecular structures and 3D models of (**A**) mTHPC and (**B**) randomly methylated β-cyclodextrin (Me-β-CDs). The degree of substitution (m = 11–14), as reported in the experimental section. (**C**) The 3D schematic representation of the stepwise formation of 1:2 inclusion host−guest complex between mTHPC and two Me-β-CDs.

Generally, PS is given by injection into a vein, named systemic administration, and its subsequent fate is determined by the binding to plasma proteins and interaction with endothelial and blood cells [2,4]. Meanwhile, many effective PSs are insoluble hydrophobic molecules tending to aggregate upon systemic administration. One such PS is temoporfin (5,10,15,20-tetrakis (3-hydroxyphenyl)chlorin, mTHPC), approved for the palliative treatment of advanced head and neck cancers in EU (Figure 1A) [5]. The mTHPC is considered as one of the most promising second-generation PSs due to its low administration dose and attractive photophysical characteristics [5,6,7]. However, high lipophilicity of mTHPC results in the formation of large non-photoactive aggregates of mTHPC in the blood, thus limiting its bioavailability [8,9]. As a consequence, mTHPC possesses a characteristic pharmacokinetic profile in humans with the delayed concentration peaks [10,11]. To avoid the precipitation of the drug and to provide its effective delivery to the target tissue, nanoscale systems for the delivery of PSs have been developed [12,13]. However, encapsulation of the drugs in nanocarriers may cause a significant impact on its interaction with blood components and subsequently on the overall distribution of the drug in the organism.

Recently, β-cyclodextrin (β-CD) derivatives were proposed as nanocarriers for mTHPC. β-CDs belong to the family of cage molecules and consist of seven D-glucopyranose units (Figure 1B) [14,15,16]. Due to the presence of a hydrophobic interior cavity, β-CDs are able to form inclusion host−guest complexes with a wide range of compounds [17,18,19], including aryl-porphyrins [14,20,21,22]. According to our data [20,23,24], β-CDs provide mTHPC solubilization by forming 1:2 supramolecular host-guest complexes with extremely high affinity (Figure 1C). The very high efficiency of the complex formation allows β-CDs to act as nanoshuttles offering unique opportunities for the delivery of mTHPC, such as alteration of the mTHPC distribution in the solution of serum proteins [25,26], accelerating the mTHPC uptake cells [26]. Moreover, β-CDs enable deep penetration of mTHPC in 3D spheroid culture cells and increase the selectivity of mTHPC uptake in xenografted tumors in vivo [26,27]. Given these particularities of β-CDs, we assumed that co-administration of mTHPC with β-CDs would significantly alter PS biodistribution in blood, particularly between blood cells.

In the present study, we evaluated the influence of randomly methylated β-CD (Me-β-CD) on the distribution of mTHPC in the whole blood of healthy donors. We assessed how the presence of Me-β-CD modulates mTHPC accumulation in the main subpopulations of blood cells. Finally, we investigated how Me-β-CD may affect mTHPC targeting of blood cancer cells.

## 2. Materials and Methods

### 2.1. Materials

The mTHPC was kindly provided by biolitec research GmbH (Jena, Germany). The stock solution of mTHPC (2 mM) was prepared in ethanol absolute and kept at 4 °C in the dark. The concentration of mTHPC in solution was estimated by spectroscopy using a molar extinction coefficient of 29,600 M^−1^ cm^−1^ at 650 nm in ethanol absolute [6]. The spectroscopy measurements were carried out using a spectrometer Solar PV 1251c (Solar, Belarus).

Randomly methylated β-cyclodextrin (Me-β-CD; CAS: 128446-36-6; substitution degree of 11–14, average molecular weight 1135 Da) was purchased from AraChem (Tilburg, The Netherlands).

### 2.2. Cell Lines

In this study, we used human peripheral blood from healthy donors. Informed consent forms were signed, as requested, and approved by the institutional review board the Belarusian Research Center for Pediatric Oncology, Hematology, and Immunology (protocol #0311; 10 November 2011). The hematocrit level of the samples was brought up to 50%. The samples were diluted five times in Roswell Park Memorial Institute 1640 medium (RPMI-1640, Invitrogen™, Carlsbad, CA, USA) and centrifuged for 15 min at 1500 rpm.

To study mTHPC uptake in the suspension of lymphocytes, they were isolated from the whole blood by centrifugation in density gradient media Histopaque-1077 (Sigma, St. Louis, MO, USA) for 25 min at 1500 rpm.

Raji (human Burkitt’s lymphoma) cell line was purchased from ATCC (Cat. No: ATCC^®^ CCL-86™). Cells were cultured in RPMI-1640 supplemented with 10% (*v*/*v*) heat-inactivated fetal bovine serum (FBS, Sigma-Aldrich, Saint-Quentin Fallavier, France), penicillin (10,000 IU), streptomycin (10,000 mg/mL), and 1% (*v*/*v*) 0.2 M glutamine (Invitrogen™, Carlsbad, CA, USA). Cells were kept as in suspension in a humidified incubator (5% CO_2_) at 37 °C. Cells were reseeded every week to ensure exponential growth.

### 2.3. Flow Cytometry

The isolated plasma was incubated with a double-concentrated mTHPC solution for 3 h to achieve complete PS monomerization. Then, 1 mL of mTHPC-enriched plasma was added to 1 mL of blood cells with/without Me-β-CD. The final concentration of mTHPC was 4.5 µM. The concentration of Me-β-CD was in the range of 3–300µM. Blood cells were incubated in the dark at 37 °C for 20 h. The samples were vortexed every 30 min during the first 4 h of incubation.

For flow cytometry analysis, 950 µL of phosphate-buffered saline (PBS; pH = 7.4) was added to the 50 µL aliquots of blood cells. After that, 100 µL of the obtained solution was incubated with 10 µL of FITC anti-human CD45 antibody solution (0.5 mg/mL) purchased from (Invitrogen™, Carlsbad, CA, USA) to distinguish white blood cells (WBC). After 5 min of incubation at 37 °C, the samples were vortexed and analyzed immediately by flow cytometry.

Flow cytometry analysis was performed using FC500 (Beckman Coulter, Brea, CA, USA), equipped with lasers emitting at 488 nm and 633 nm. The fluorescence of CD45-FITC was detected in the fluorescence channel FL1 with a 525 ± 15 nm filter under the excitation at 488 nm, while the detection of mTHPC fluorescence was performed in FL4 channel with 675 ± 15 nm filter under the excitation at 633 nm. Data analysis was carried out using CXP Software (version 2.2, Beckman Coulter, Brea, CA, USA). Uptake of mTHPC was presented as mean fluorescence intensity in arbitrary units (a.u.).

The isolated lymphocytes were twice washed in PBS and incubated with mTHPC (4.5 µM) in RPMI-1640, supplemented with 2% FBS for 3 h at 37 °C.

To study uptake in Raji cells, the culture medium was replaced by RPMI-1640, supplemented with 2% FBS pre-incubated with (4.5 µM) mTHPC for 3 h at 37 °C. Then, 1–100 µM of Me-β-CD was added to the samples. Samples were incubated in the dark at 37 °C for 24 h. The final concentration of cells was 10^6^ cells/mL. For kinetic measurements, 50 µL aliquots were taken from each sample and analyzed by flow cytometry.

### 2.4. Me-β-CD Toxicity

To assess Me-β-CD-induced hemolysis, the whole blood was diluted five times with PBS, centrifuged for 10 min at 1500 g, and the supernatant was removed. These steps were repeated three times. The obtained cell suspension was diluted 10 times in PBS and incubated with 300, 600, 1000, 2000, 4000 and 5000 µM of Me-β-CD and without Me-β-CD (negative control; 0% hemolysis). Additionally, the cell suspension diluted 10 times in distilled water was taken as a positive control (100% hemolysis), as described elsewhere in [28]. After 2 h at 37 °C, the samples were centrifuged, and the supernatant was analyzed spectroscopically at 405 nm. The data are presented as a percentage compared to the positive control sample.

The viability of WBC in the presence of 10, 50, 100, 500 and 1000 µM of Me-β-CD was assessed using propidium iodide (PI), membrane impermeant dye, at 20 h post-incubation. Cells were incubated in RPMI-1640 media supplemented with 2% FBS for 20 h at 37 °C. WBC incubated in Me-β-CD-free media were taken as a negative control. The cell suspension was stained with 1 µg/mL PI (Biolegend, San Diego, CA, USA) for 15 min at room temperature and analyzed by flow cytometry. PI fluorescence was detected in FL2 with a 575 ± 15 nm filter (excitation at 488 nm).

### 2.5. Fluorescence Microscopy

The analysis of intracellular localization of mTHPC in Raji cells was performed using laser confocal scanning microscope Leica DM2500 (Leica microsystem, Wetzlar, Germany). Raji cells were incubated with (4.5 µM) mTHPC for 3 h at 37 °C in RPMI-1640 media supplemented with 2% FBS and with/without Me-β-CD (1, 20, 50 µM). The cell suspension was washed twice after incubation with mTHPC. Then, the cells were placed on the SuperFrost glass, covered by the thin glass (0.17 mm), and analyzed by confocal microscopy (×40 air objective and ×63 oil immersion objective). The fluorescence images were obtained using 532-nm excitation and registration at 630–680 nm. The analysis of images was performed with ImageJ (NIH, Bethesda, MD, USA) software (version 1.53).

### 2.6. Statistics

The data from at least three independent experiments are presented as mean ± standard deviation (SD). An unpaired, two-tailed t-test was used for statistical analysis of two groups with a significance level of *p* < 0.05. Analysis of Variance (ANOVA) followed by Tukey’s multiple comparisons test was used for comparison of three or more groups. Data analysis was carried out with the Origin software (version 9.0, OriginLab, Northampton, MA, USA).

## 3. Results

### 3.1. Distribution of Free mTHPC in Blood

**Figure 2:**
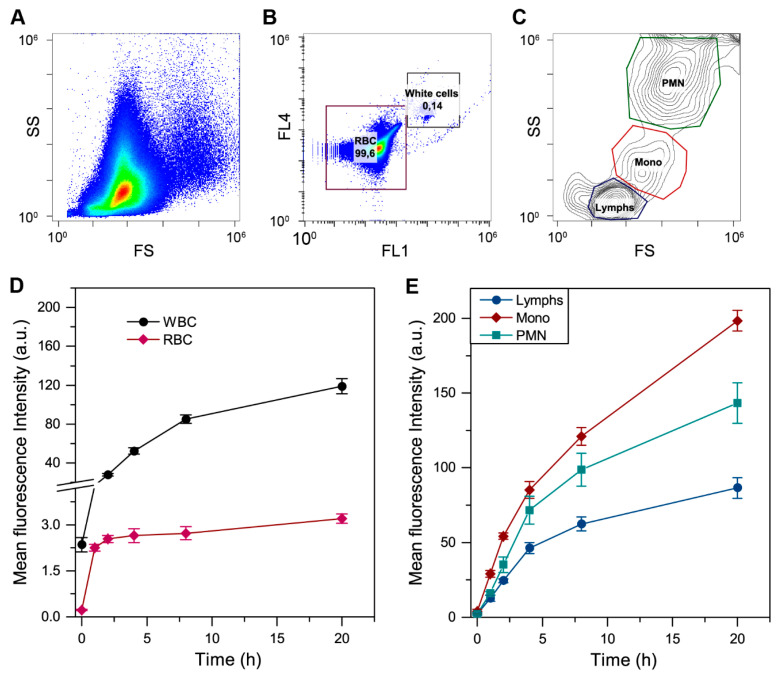
Flow cytometry analysis of mTHPC distribution in blood components. (**A**) Side scatter (SS)/forward scatter (FS) dot plot of blood cells population. (**B**) Separation of RBC and WBC populations using CD-45 antibody (FL1). (**C**) Separation of white blood cells on Lymph (blue), Mono (red) and PMN (green) subpopulations based on SS/FS characteristics. (**D**) Uptake kinetics of mTHPC in RBC (♦) and WBC (●) cells. (**E**) Uptake kinetics of mTHPC in Lymps (●), Mono (♦), and PMN (■) cells. Graphs represent the mean ± SD. The concentration of mTHPC was 4.5 µM.

To evaluate the distribution of Me-β-CD-free mTHPC in subpopulations of blood cells, we analyzed the blood incubated with mTHPC by flow cytometry. The typical scattering dot plot (FS~SS) is displayed in Figure 2A. To distinguish red blood cells (RBC) and white blood cells (WBC), the samples were incubated with CD45-FITC antibody (FL1), which is selective for WBC (Figure 2B). Typically, in healthy subjects, the fraction of WBC in the blood does not exceed 0.2%. Therefore, we analyzed over 10^6^ cells per sample to achieve a detectable amount of WBC. Finally, the subpopulations of lymphocytes (Lymph, blue contour), monocytes (Mono, red contour), and granulocytes (namely, polymorphonuclear leukocytes, PMN, green contour) were distinguished using their difference in scattering properties (Figure 2C), as was reported earlier [29]. The fractions of Lymph, Mono, and PMN were 64 ± 10%, 11 ± 5%, and 25 ± 10% of WBC, respectively.

The kinetics of mTHPC uptake in particular subpopulations of blood cells is presented as mean fluorescence intensity (MFI) (Figure 2D,E). In fact, mTHPC accumulation in RBC reaches the plateau at 1 h and does not change until 20 h of incubation. Meanwhile, WBC intensively accumulate mTHPC, and after 20-h incubation, MFI of WBC was almost 40 times higher compared to RBC (119 a.u. vs. 3.2 a.u.). In particular, mTHPC uptake was increasing in the following order: Lymps < PMN < Mono (Figure 2E), reaching MFI of 87, 143, and 198 a.u. at 20-h incubation. 

### 3.2. Alteration of mTHPC Distribution in Blood by Cyclodextrins

#### 3.2.1. Me-β-CD Toxicity

**Figure 3:**
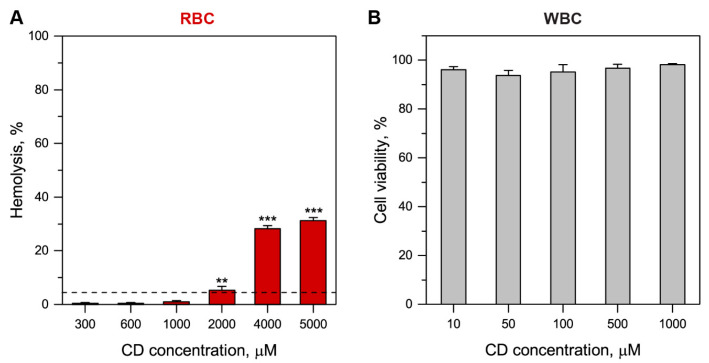
Cytotoxicity of Me-β-CD. (**A**) Hemolysis assay and (**B**) WBC viability in the function of Me-β-CD concentration. Hemolysis was analyzed for 2-h incubation with Me-β-CD, while the WBC viability was assessed using PI at 20 h post-incubation. Data represent averages ± SD (*n* = 3; ** *p* < 0.01, *** *p* < 0.001 using the one-sample t-test (µ = 0)). Dashed line corresponds to 5% of hemolysis.

In order to assess Me-β-CD toxicity towards RBC and WBC, we used hemolysis assay and PI staining of necrotic WBC (Figure 3). According to the obtained data, the detectable hemolysis of 5% was observed 2 h post-incubation with 2 mM of Me-β-CD (Figure 3A). The presence of 4 mM and 5 mM of Me-β-CD resulted in 28% and 31% of hemolysis, respectively. At the same time, WBC cells were viable after 24-h incubation in the presence of 1 mM Me-β-CD (Figure 3B). Cell viability of WBC incubated for 20h without Me-β-CD was 97%. In summary, Me-β-CD concentrations below 1 mM could be considered non-toxic and were used for further experiments.

#### 3.2.2. Redistribution of mTHPC from Plasma to Blood Cells

**Figure 4:**
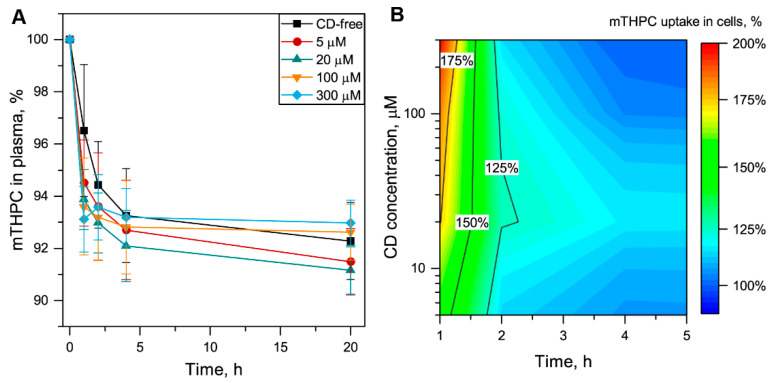
Redistribution of mTHPC from plasma to the blood cells in the presence of Me-β-CD. (**A**) Kinetics of mTHPC redistribution from plasma to the blood cells in the absence of Me-β-CD (■) and in the presence of 5 µM (●), 20 µM (▲), 100 µM (▼), and 300 µM (♦) of Me-β-CD. Graphs represent the mean ± SD (*n* = 3). (**B**) The 2D contour plots of mTHPC uptake in blood cells in the function of time and concentration of Me-β-CD. Uptake of mTHPC in the absence of Me-β-CD was taken as 100%. The concentration of mTHPC was 4.5 µM.

To evaluate the influence of Me-β-CD on mTHPC distribution in blood, we estimated the kinetics of mTHPC redistribution from plasma proteins to blood cells. In particular, we incubated mTHPC with isolated plasma for 3 h to avoid mTHPC aggregation, and then mixed mTHPC-enriched plasma with blood cells in the presence of various Me-β-CD concentrations. Then, we again isolated the plasma and spectroscopically estimated the amount of mTHPC bound to plasma proteins (Figure 4A). In Me-β-CD-free conditions, 92% of mTHPC was detected in plasma at 20 h post-incubation with blood cells. It means that only 8% of PS from serum redistributed to the cells. Additionally, Me-β-CD altered mTHPC redistribution from plasma proteins to the blood cells. In fact, in the presence of 5–20 µM of Me-β-CD, blood cells accumulated a bit more PS (9%), while at 300 µM, the amount of mTHPC, redistributed from plasma was 7%. To visualize the influence of Me-β-CD on the amount of mTHPC redistributed to the blood cells, we normalized the data to the Me-β-CD-free sample (100%) and plotted 2D contour plots of mTHPC uptake in blood cells in the function of both Me-β-CD concentration and time (Figure 4B). We demonstrated that all studied Me-β-CD concentrations resulted in acceleration of mTHPC redistribution during the first 2-h incubation. The maximal values corresponding to 300% acceleration of cellular uptake were obtained for 300 µM Me-β-CD at 1 h post-incubation. We observed that such high Me-β-CD concentration resulted in the slowdown of mTHPC uptake in blood cells. It is worth noting that the spectroscopy approach allowed us roughly to determine the fraction of the concentration of mTHPC uptaken by the cells; however, spectroscopy approach was limited in sensitivity and did not allow investigation of PS accumulation in subpopulations of blood cells. Thus, further experiments were carried out using flow cytometry.

#### 3.2.3. The mTHPC Accumulation in Subpopulations of Blood Cells

**Figure 5:**
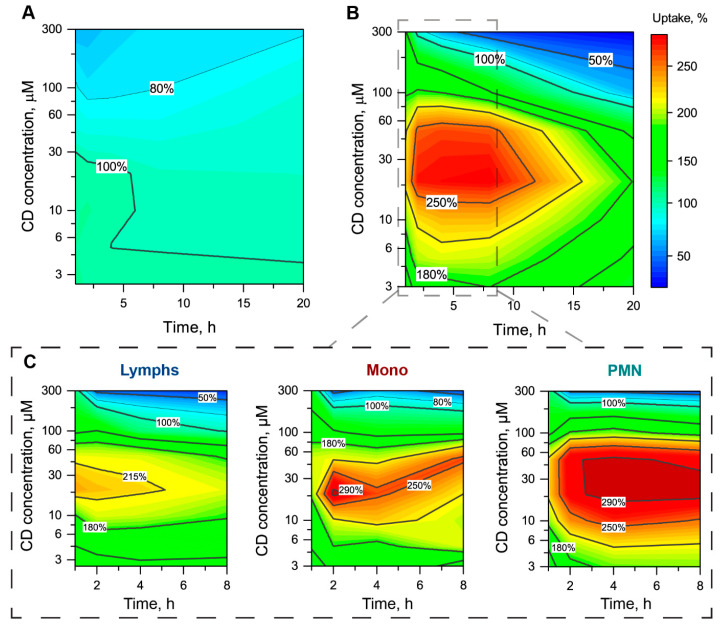
Alteration of mTHPC uptake in different populations of blood cells in the presence of Me-β-CD. The 2D contour plots of mTHPC uptake in (**A**) RBC, (**B**) WBC, and (**C**) particular WBC populations (Lymps, Mono, PMN) as the function of time and concentration of Me-β-CD. Uptake of mTHPC in the absence of Me-β-CD was taken as 100%. The concentration of mTHPC was 4.5 µM.

We analyzed in detail the alteration of mTHPC accumulation in individual blood cells by Me-β-CD. The obtained data were normalized to the Me-β-CD-free sample (100%) and plotted as 2D contour plots of mTHPC uptake kinetics in the function of Me-β-CD concentration for each subpopulation of blood cells (Figure 5). We demonstrated that Me-β-CD slightly affected mTHPC accumulation in RBC (Figure 5A). In particular, the addition of Me-β-CD at concentrations up to 100 µM did not change the uptake of mTHPC in RBC. Further increase of Me-β-CD concentration resulted in the gradual decrease of MFI to 62–78% for 300 µM at 1–20 h.

Meanwhile, in the case of WBC, the presence of Me-β-CD strongly modulated mTHPC accumulation (Figure 5B). Generally, the addition of Me-β-CD in the range of 3–100 µM led to the significant acceleration of mTHPC uptake in WBC. The maximal acceleration of 280% was observed for 20 µM of Me-β-CD in the medium during the first 8 h of incubation. The analysis of mTHPC uptake in the subpopulation of WBC showed the increased acceleration in the following order: Lymps > Mono > PMN (Figure 5C). Indeed, mTHPC accumulation in PMN was almost three times higher in the presence of Me-β-CD concentrations, ranging from 20–50 µM at 1–8 h, compared with Me-β-CD-free samples. For Mono, the maximal mTHPC uptake (300% compared to Me-β-CD-free sample) was observed at 2-h incubation in the presence of 20 µM Me-β-CD. Finally, Lymps were less sensitive to the presence of Me-β-CD. The maximal mTHPC uptake was 230% at 1-h incubation.

It is worth noting that Me-β-CD at high concentrations (over 200 µM) could also inhibit mTHPC uptake in WBC, especially for long incubation times (more than 5 h). Among WBC subpopulations, the strongest inhibition of mTHPC accumulation was observed for Lymps (32% of control) at 300 µM of Me-β-CD and 8 h post-incubation.

### 3.3. Me-β-CD-Modulated mTHPC Accumulation in Blood Cancer Cells

**Figure 6:**
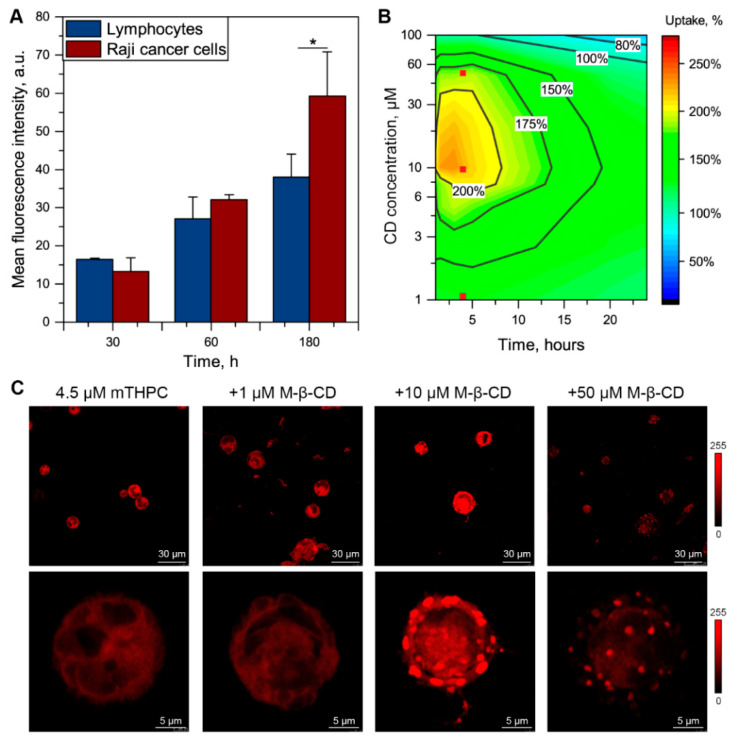
Alteration of mTHPC uptake in Raji cells in the presence of Me-β-CD. (**A**) Uptake of mTHPC in patient-derived lymphocytes ( █ ) and Raji ( █ ) cancer cells after 0.5-h, 1-h and 3-h incubation. Graphs represent the mean ± SD (*n* = 3; * *p* < 0.05, using two-sample t-test). (**B**) The 2D contour plot of mTHPC uptake in Raji as the function of time and concentration of Me-β-CD. Uptake of mTHPC in the absence of Me-β-CD was taken as 100%. Red dots (■) correspond to the conditions chosen for microscopy analysis. (**C**) The typical fluorescence images of Raji cells exposed to mTHPC for 3 h in the absence/presence of various Me-β-CD concentrations; ×40 air objective and ×63 oil immersion objective. High-magnification images display the surface of the cells. The concentration of mTHPC was 4.5 µM. Serum concentration was 2%.

In addition, we evaluated the influence of Me-β-CD on the intracellular accumulation of mTHPC in human Burkitt lymphoma Raji cells. For this purpose, we incubated the suspension of Raji cells with 4.5 µM mTHPC in the presence of various concentrations of Me-β-CD. Firstly, we compared cellular uptake of mTHPC in Lymps, isolated from the blood of healthy patients, and Raji cancer cells in Me-β-CD-free conditions (Figure 6A). In fact, Raji better accumulated mTHPC than Lymps from the blood of healthy patients after 3-h incubation (*p* > 0.05). Of note, at 0.5-h and 1-h incubation, no significant difference in PS uptake was observed between normal Lymp and Raji cancer cells.

In the presence of Me-β-CD, we observed the alteration of mTHPC accumulation kinetics in Raji cells (Figure 6B). The strongest acceleration of PS uptake was detected for 8–40 µM of Me-β-CD during the first 6 h of incubation. Additionally, slight inhibition of uptake was observed 8 h post-incubation for Me-β-CD concentrations over 80 µM (80% compared to Me-β-CD-free condition). In addition, we assessed intracellular localization of mTHPC in Raji cancer cells using laser confocal scanning microscopy (LCSM) in the presence of 1, 10, and 50 µM of Me-β-CD at 4-h incubation (Figure 6C). The chosen conditions are marked as red dots on 2D contour plot in Panel B of Figure 6. In the Me-β-CD-free medium, mTHPC was evenly accumulated in the cytoplasm of Raji cells. In the presence of low Me-β-CD concentration (1 µM), no visual difference was observed. Meanwhile, the addition of 10 µM resulted in evident changes in the intracellular localization of mTHPC. Indeed, there was a spotted pattern of mTHPC distribution, preferably on the periphery of cells in the plasma membrane compartment. Further increase in Me-β-CD concentration up to 50 µM led to the similarly heterogeneous mTHPC fluorescence pattern in the Raji cells, but with noticeably lower overall fluorescence, thus confirming the data obtained by flow cytometry.

## 4. Discussion

The results obtained in this study suggest that we could modulate the cell-bound fraction of mTHPC in blood by varying the concentration of Me-β-CD. The present study is a continuation of our recent research on the application of β-cyclodextrins monomers as nanocarriers for mTHPC delivery in vitro [23,27] and in vivo [26]. Here, we studied the biodistribution of mTHPC/β-CD complexes ex vivo on blood samples of healthy donors as a more relevant model of drug interaction with components of human blood compared to the rodents. We demonstrated, that Me-β-CD is well-tolerated and exhibited limited cytotoxicity and hemolysis at high concentrations (up to 2 mM). It was shown, that mTHPC accumulation in WBC strongly depends on Me-β-CD concentration and incubation time, while RBC uptake of mTHPC was affected by only high concentrations of Me-β-CD (up to 100 µM). Furthermore, we showed the alteration of uptake and intracellular localization of mTHPC in Burkitt′s lymphoma Raji cells in the function of Me-β-CD concentration.

As mTHPC is a highly lipophilic drug molecule (log P = 8.8; Pubchem database, CID 60751), it tends to be incorporated into the lipid structures (e.g., lipoproteins, cellular lipid membranes). Thus, the processes of interaction with serum proteins and blood cells are important for the understanding of mTHPC biodistribution and pharmacokinetics. Though several studies investigated the distribution of mTHPC between serum proteins [8,30], relatively little attention has been paid to the cellular uptake of mTHPC in blood cells. In particular, the recent study of Jablonka and co-workers considered a cell-bound fraction of mTHPC simulating human biodistribution and pharmacokinetics [9]. However, their considerations were limited to monocytes. In the present study, we demonstrated that monocytes better accumulate mTHPC than other types of blood cells; however, the observed difference in cellular uptake did not allow us to neglect the fractions of PS bound with Lymps and PMN. On the other hand, WBC cells accumulated 40 times more mTHPC than RBC. We supposed that such poor uptake in RBC is related to their morphology and lack of cell nuclei and most membranous organelles [31]. Nevertheless, given that the fraction of WBC in the blood does not exceed 0.2%, we could not neglect RBC-bound mTHPC, since the total PS fraction in RBC will be higher than that in WBC.

β-CDs have been widely investigated as a unique pharmaceutical excipient for the past few decades and they are still explored for new applications [14,15]. β-CDs are considered as a promising drug delivery system for PS [14,32,33]. The formation of inclusion complexes increases the solubility of highly hydrophobic PS and their physical and chemical stability [32,34]. Recent reports demonstrated the potency of β-CDs as an individual nanocarrier of mTHPC in in vitro and in vivo preclinical tumor models [26]. Deep studies of β-CDs as pharmaceutical agents demonstrated that β-CDs effect strongly depends on β-CDs affinity to the drug [15]. Several reports demonstrated that β-CDs may alter cellular uptake of aryl-porphyrins [35,36,37], including mTHPC [26]. Previously, we studied the mechanism of formation of inclusion complex between β-CDs and mTHPC, using optical techniques of molecular dynamics modeling [20,24]. According to our calculations, randomly methylated Me-β-CD exhibited a very high affinity to mTHPC (K = 7.1×10^5^ M^−1^), forming a 1:2 inclusion complex (Figure 1). In turn, such high binding affinity provides the unique opportunity to modulate PS behavior in biological media [13]. Earlier, we demonstrated the alteration of mTHPC distribution in serum in the presence of β-CDs [26]. In fact, both concentration and type of CDs play key roles in the alteration of mTHPC distribution. β-CDs act as temporary transporters of PS molecules between plasma proteins, while in the presence of the excess of β-CDs molecules they act as competitive binding sites. Hence, the equilibrium distribution of PS in serum shifts to the inclusion complexes resulting in the sequestration of mTHPC in inclusion complexes. Nevertheless, the distribution between the main serum proteins (e.g., albumin, high-density lipoprotein and low-density proteins) remains constant. Importantly, in the presence of many potential binding sites, the probability of the formation of complexes is determined by the binding constant of CDs to mTHPC.

In the present work, we demonstrated that the addition of Me-β-CD to the blood affects the accumulation of mTHPC in blood cells, particularly in WBC, due to the higher volume of membranous organelles compared to RBC. Different subpopulations of WBC exhibit various sensitivity to the modulation of mTHPC uptake by Me-β-CD, increasing in the following order Lymps > Mono > PMN (Figure 5). Meanwhile, the maximal acceleration was displayed for 20–50 µM of Me-β-CD at 2–4 h post-incubation for all types of WBC. These results encouraged us to study in detail how Me-β-CD modulates the accumulation of mTHPC by cancer human Burkitt′s lymphoma Raji cells. As seen in Figure 6, the addition of Me-β-CD at 10 µM resulted in two-times higher mTHPC cellular uptake in “spots” in the plasma membrane compartment. Spotted localization pattern could be attributed to the preferable accumulation of mTHPC in cholesterol-depleted areas of the plasma membrane, formed by Me-β-CD, as was reported in our earlier work on monolayer cancer cells [26].

## 5. Conclusions

Overall, our study clearly demonstrated that β-CDs derivatives provide new options to modulate mTHPC biodistribution in the course of PDT. We showed that cell-bound mTHPC fraction in blood strongly depends on the concentration of Me-β-CD. In fact, the accumulation of mTHPC in WBC was more sensitive than that in RBC. Furthermore, one could expect that Me-β-CD could modulate the cellular uptake in blood cancer cells. Given the increasing interest in β-CDs as a component of hybrid nanocarriers (e.g., drug-in-cyclodextrin-in-liposome [38,39,40], cyclodextrin-based polymers [41]), the detailed analysis of mTHPC distribution provides a better understanding of how we can alter biodistribution and pharmacokinetics of mTHPC using nanotechnology aiming for a high therapeutic efficiency of mTHPC-PDT with low side effects.

The results obtained in the present work will be useful to study the relationships between the interaction of drug with blood components, accumulation of the drug in endothelium cells, and penetration of drug into the tumor parenchyma.

## Data Availability

The data sets used and/or analyzed during the current study are available from the corresponding author on reasonable request.
